# Temporal Vulnerability and the Post-Disaster ‘Window of Opportunity to Woo:’ a Case Study of an African-American Floodplain Neighborhood after Hurricane Floyd in North Carolina

**DOI:** 10.1007/s10745-017-9915-4

**Published:** 2017-07-17

**Authors:** Daniel H. de Vries

**Affiliations:** Department of Anthropology, University of Amsterdam, Nieuwe Achtergracht 166, 1018 WV Amsterdam, The Netherlands

**Keywords:** Temporality, Vulnerability, Surprise, Referentiality, United States, North Carolina, Hurricane Floyd

## Abstract

After major flooding associated with Hurricane Floyd (1999) in North Carolina, mitigation managers seized upon the “window of opportunity” to woo residents to accept residential buyout offers despite sizable community resistance. I present a theoretical explanation of how post-crisis periods turn into “opportunities” based on a temporal referential theory that complements alternative explanations based on temporal coincidence, panarchy, and shock-doctrine theories. Results from fieldwork conducted from 2002 to 2004 illustrate how several temporal influences compromised collective calibration of “normalcy” in local cultural models, leading to an especially heightened vulnerability to collective surprise. Four factors particularly influenced this temporal vulnerability: 1) epistemological uncertainty of floodplain dynamics due to colonization; 2) cultural practices that maintained a casual amnesia; 3) meaning attributed to stochastic timing of floods; and 4) competitive impact of referential flood baseline attractors.

## Introduction

In 1999, a $40 million home acquisition and resident relocation Hazard Mitigation Grant Program (HMGP) was sponsored by the United States Federal Emergency Management Agency (FEMA) to buy residents out of the floodplain of the small city of Kinston in eastern North Carolina (pop. 25,000). In total this program moved 4% of nearly 10,000 City households, many from a prominent, historically African-American neighborhood referred to as “Lincoln City.” Because 95% of property owners who received a buyout offer accepted, the buyout was considered immensely successful and had been heralded as a model floodplain mitigation program (NCDEM 1999; FEMA 2003, 2004, 2007; Olivera-McCan 2006). Yet FEMA evaluation data including the Kinston buyout only partly confirm this celebratory view, reporting a consistent perception of involuntariness among approximately one-third of the sample (Fraser *et al.* 2003). Further quantitative and qualitative analysis of this dataset found that this attitude of resistance was not statistically related to people leaving against their will in the face of high perceived risk of repeat flooding nor to issues of community attachment, but instead to local government strategies and tactics (De Vries and Fraser 2012).

Federal eligibility requirements for FEMA post-disaster grants that require “strictly voluntary” participation raised the question of how this situation of involuntary agreement to relocate out of the floodplain developed. My initial thesis included the possibility it was an issue of environmental injustice within a (still) racialized southern U.S. context. This explanation however fails to do justice to the best intentions of local mitigation managers and officials trying to restore normalcy and prevent future risk. As I continued my fieldwork, another conclusion emerged that the situation had developed as a result of a “window of opportunity” recognized by local emergency managers who noted the situation as the right moment to implement a controversial mitigation agenda (De Vries and Fraser 2012). During one interview with FEMA officials in Washington D.C. this intervention strategy was emphasized as a common practice (even though it was quickly noted that it may need some reform): “I think often post-disaster decisions can be made by capitalizing on a loss of taking advantage of people…we used to call it ‘the window of opportunity to woo’ [laugh]”.

Despite common acknowledgment in practice, there has been relatively little theoretical exploration of the post-crisis “window of opportunity” for change (Christoplos 2006). In an organizational context, based on Schein’s (1972) idea that crises may be “unfreezing events,” Carley and Harrald suggest that this “window of opportunity” is based on a “temporary staggering of basic assumptions” that allows for change from pre-established patterns (Carley and Harrald 2008:107). They note that this crisis-induced “thaw” is of limited duration and after the “window” closes change is less likely, thus implicitly focusing on the temporary loss of normalcy among those undergoing the crisis. Yet, within global change literature the few theorists who have directly addressed this issue take a more socio-political perspective (Birkmann *et al.* 2010; McSweeney and Coomes 2011; Manyena 2013). For example, McSweeney and Coomes focus on how during the “window of opportunity” post Hurricane Mitch in Honduras the poor were able to mobilize political forces to initiate institutional change and increase resilience, e.g., through relocation of production away from risky floodplains, renewal of social cohesion through more equitable intra-community distribution of land, and by restoration of more diverse income-generation strategies. While they provide historical insight into the impact of socio-political factors, their approach seems to presume that all post-disaster “windows of time” are similar in character. The difference between the outcomes of “opportunity lost” or “opportunity used” appears here dependent mostly on the historical ability of involved social movements to effectively manipulate socio-political processes. However, McSweeney and Coomes’ analysis does not include issues related to cultural normalcy linked more directly to the temporary staggering of basic assumptions. While they provide historical analysis of root vulnerabilities, they do not address how resilience of Hondurian communities after Hurricane Mitch may also be related to of this population’s experience with a series of previous hurricane events in 1961 (“July 23rd”), 1966 (“Alma”), 1969 (“Francelia”), 1971 (“Sept 9th”) and 1974 (“Fifi”), and 1978 (“Greta”).

Rather than inquiries into what unfreezes “the normal,” other explanations for the agentive power of the “window of opportunity” resemble McSweeney and Coomes’ analysis by focusing on what happens during the “window.” Birkmann *et al.* (2010), citing Kingdon (1995), provide a theory of temporal coincidence to argue that “windows of opportunity” emerge because separate streams of problems, policies, and politics converge to produce a critical time where solutions become joined problems. This perspective is reminiscent of the socio-ecological theory of *panarchy* that is based on the idea that risks organize at multiple temporal scales (Bankoff *et al.* 2004), but may develop into a critical time when dynamics in different temporal scales overlap chronologically, reinforcing each other and leading to sudden cross-scale, “panarchic” revolts (Gunderson and Holling 2002; Gallopı 2006). Finally, an explanatory “shock-doctrine” thesis for the “window of opportunity” has been popularized by the investigative journalist Naomi Klein. Building on age-old military strategies, Klein argues that a “critical time” may be politically manipulated by imposing an event that holds a shock value. She argues that Milton Freedman’s awareness of a critical post-crisis opportunity for economic change led him to support strategies of state-induced terror or exploitation of natural disasters in order to enable the passage of controversial, exploitative policies at a time when citizens are too preoccupied by these upheavals to create an effective resistance (Klein 2007).

These theories have a common perspective that focuses on the description of what happens *during* the temporal window. As such, these perspectives obscure the possibility that the historical and temporal dynamics leading up to the critical time may have already conditioned—facilitated or constrained—the extent to which a positive or negative outcome could develop at all. These perspectives, in other words, do not provide an emic perspective conceptualizing how the unfreezing of basic assumptions among stakeholders may influence the “leniency” of the “window of opportunity” itself.

## Temporal Referentiality

Here, I elaborate on a previously outlined theory (De Vries 2007, 2008) to explain the agentive power of the window of opportunity based on an analysis of temporal referentiality in cultural models. Cultural models—although somewhat outdated when associated only to notions of Culture as static—can be seen as dynamic explanatory systems that connect parts and emulate relationships among mental constructs. They enable prediction and explanation, and are cultural because they are shared and reproduced within a culture (Holland and Quinn 1987). By necessity, the representation of the environment in cultural models must include historical ecological knowledge that orients expectations to what “normal” system (e.g., floodplain) behavior may look like (Crumley 1994, 2002). This includes the recognition of temporal features in the changing landscape and other forms of historical knowledge such as intergenerational memory. This type of knowledge arguably exists in the present through temporal references that calibrate meaning to past experiences (De Vries 2011a). Cultural models integrate broader presumptions about social reality, such as the sociological framing of risk-society (Beck 1992), but also the particularities of local cultural histories.

From this cultural model perspective, the “window of opportunity” may become a vulnerability to change—or have increased agentive power to be an “opportunity” at all—when temporal referentiality becomes compromised. As argued previously, the resulting “temporal vulnerability” (De Vries 2008, De Vries 2011a, 2011b) is expressed through the experience of collective surprise, of which the logical consequence would be an unfreezing of long-standing expectations (De Vries 2007, 2008, 2011a, 2011b). Holling (1986) has characterized surprise from an ecological perspective as occurring when causes turn out to be sharply different than conceived, when behaviors are profoundly unexpected, and when action produces a result opposite to that intended—in short, when perceived reality departs qualitatively from expectations. Because there can be no informed expectation without time series data, cultural memory or personal experience, this vulnerability is temporal; dealing with our being in time, timing and history itself, and should be based on an analysis of derived human expectations. This not to establish the notion that reality is a social construct or that baselines depend on cultural memory (e.g., Hulme *et al.* 2009), but rather to investigate how these temporal constructions produce vulnerability to change.

The temporal vulnerability framework provides a direct explanation for the emergence of critical times based on the notion that the issue of causal concern is time and timing, not as background but as cause, with a key role of history itself through the mediation of cultural models and embedded temporal referentiality, including social time. This is different from the coincidence and panarchic theories noting that critical time is caused by multiple socio-political factors or temporal scales compounding vulnerability in a certain moment of time (Moseley 1999, Moseley 2002; Dyer 2002). In these conceptualizations, time is still seen as a chronic state of affairs, a timeline against which pressure builds up. While it is crucial to acknowledge that vulnerability changes through time in unpredictable ways and in varying directions (Bankoff *et al.* 2004:6), these are still claims about historical analysis of vulnerability of any type, but not about the *human experience of time* rendering certain periods critical, and others not. To understand critical time, I believe a subjective and foremost cultural qualification has to complement other perspectives to take into account our experience of reality, referred to as “social time,” a time which is fundamentally rooted in qualitative understanding (Ingold 1993), grounded in the “rhythms, pulsations, and beats of the societies in which they are found” (Sorokin and Merton 1937: 623).

Boonstra and De Boer (2014) in their analysis of socio-ecological traps claim that these situations are “causally produced through a conjunction of events” (p.26). The distinction between social time and chronological time has however rarely been mentioned by the hazard community (Forrest 1993; Alexander 2000; Bankoff 2004; National Research Council 2006). Bankoff (2004) uniquely examined the possibility that time itself may contribute to vulnerability, posing a

… temporally produced state of vulnerability… In other words, history also generates its own form of vulnerability that in a real sense underlies all other forms of vulnerability though its recognition as a factor is always more implicit than explicit. First, of course, there is the particular sequence of events that situate people in time and place; then there are the historical processes that determine their condition and their capacity to withstand its effects. But individuals also ‘construct’ disasters as both a function of their prior experience of hazards as well as from their particular ‘class’ or social group’s perception of what is happening around them (Hilhorst 2004). Moreover, disasters are not so much objective events as subjective ones that can be privileged or erased according to a sense of selective memory or collective amnesia (p.34).

Bankoff argues that time itself is as much a factor needing consideration in how disasters are created, as are politics, society, the economy, culture, and the environment. Several other authors identify indirectly the notion that there may be something important to the idea that social time and vulnerability are connected, with attention to issues of cultural memory (Hulme *et al*. 2009; Ratter 2013; Boonstra and De Boer 2014). Outside disaster literature, “vulnerable times” are typically conceptualized more simply as moments when hazard events impact systems during heightened vulnerability (West 1987; Maier *et al.* 1997; Tolich 2004; Tolich and Baldwin 2005). The added value of this study is to theorize critical time from a vulnerability perspective and investigate the historical root causes of windows of opportunity. The general vulnerability to hazards perspective within disaster research allows for thinking about factors that enhance the potentialities for loss (Torry 1979; Oliver-Smith 1996; Cutter 1996, Cutter *et al.* 2003).

To illustrate this perspective, I trace 100 years of flood and socio-political history of Lincoln City. This approach largely follows the argument made by disaster theorists that vulnerability analysis should consist of rigorous historical analysis aimed at understanding causal or root conditions of disasters (Garcia-Acosta 2002:66; Hoffman and Oliver-Smith 2002; Wisner *et al.* 2005). The case study documents how a “window of opportunity” emerged at the end of this period in 1999 favoring buyout mitigation and illustrates how challenges related to temporal referencing of past flood experiences influenced the course of events during the “window of opportunity.” I thus show how the “window of opportunity” to change can conceptually also be seen not just as a historically disconnected social dynamic, but as a systemic vulnerability with historical root causes that deals with the lived-experience of “being in time.” I have argued elsewhere that such temporal vulnerability (De Vries 2007, 2008, 2011a, 2011b) strongly influences the size of the “opportunity field” (or leniency) that exists within the post-crisis experience, and is an influence co-creating “critical time.”

## Methods

Intermittent ethnographic fieldwork was conducted between March 2002 and August 2005, with short-term visits to the study site on a monthly basis on average. I conducted interviews and documentary research at the archives of the Kinston Free Press at Kinston’s Lenoir College. In total, I conducted 50+ informal conversations in various settings and repeated open-ended interviews (average length of two hours) with 18 key informants associated with the minority neighborhood of Lincoln City. Most informants were former residents, with the exception of two lead mitigation officials, and one key mitigation consultant. All interviews were held with informed consent. Questions were focused on historical knowledge and experiences with the local flood ecology, as well as degrees of surprise (“did the flood surprise you?”). In addition, five semi-structured qualitative interviews (average length of 1.5 h) with mitigation officials from the city and surrounding County from the original buyout study (Fraser *et al.* 2003) were incorporated for further analysis. These interviews included questions about the people and organizations involved in conducting each buyout program, perceptions as to how the buyout process worked, and recommended improvements for buyout program participation. All data were organized using Nvivo qualitative software for transcribed interviews. Temporal references and different conditions that sustained temporal orientations were identified, thematically clustered, and analyzed. The methodological emphasis in this analysis is on the *quality* of the linkages between cultural expectations and biophysical reality, which can be made visible by methodologically tracing temporal references*,* or how respondents, documents, or other networks of information bring back the past in narratives (De Vries 2011a, 2011b). I critically evaluated the quality of these chains of referential transformations (Latour 1999) to assess how and to what extent they provide conditions for population surprise.

## Results

### Early Colonization of the Floodplain

**Fig. 1:**
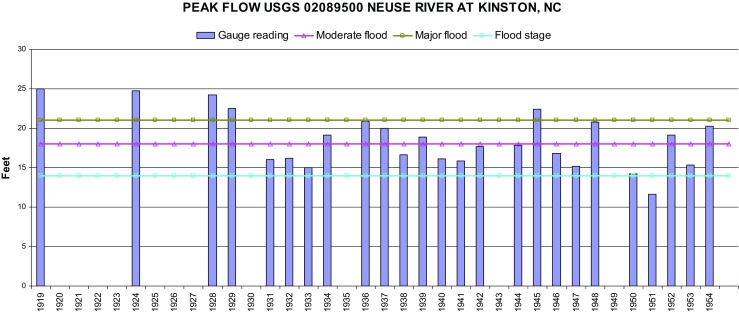
USGS gauge #02089500 Neuse River at Kinston 1919 through 1964

After initial colonization of the forested lowlands in the south of the City of Kinston by an educated black man and son of a preacher (Lincoln Barnette), a place of spiritual and recreational needs developed for the black community near the Neuse River bank named Lincoln City (Murphy 2012). Located in what only many years later would come to be formally acknowledged as the Neuse River 100-year floodplain, elderly residents I spoke to noted that their parents consistently referred to the neighborhood of Lincoln City as the “bottoms” or “lowlands” since “water always stood out there.” Oral histories suggest that in the 1930s flooding was a seasonal problem, reoccurring anytime the Neuse River was higher than 14 ft or with rainstorms. The Kinston Free Press (1996d) reported a string of flood events that affected the City in 1908, 1919, 1924, 1928, and 1945. Drainage ditches, retaining walls, and other protective structures characterized the Lincoln City landscape and residents learned to not plant too many gardens. Despite a number of moderate to major floods recorded since 1918 by the United States Geological Survey (USGS) (gauge #02089500) at the City of Kinston (Fig. 1), excluding large floods in 1908 and 1913 that occurred prior to the baseline value for the dataset, elderly respondents did not recall their parents talking much about flooding, although conversations about river heights did occur.

One key informant explained that concerns were generally not high because the flood hazard was not perceived to be life threatening. Yet, the racial composition of Lincoln City suggests that this attitude had a deeper cultural logic. Two informants explained:

It was an opportunity to have a decent place to live. That was their main, main concern. They did not ask any questions. Nobody told them anything, so they did not ask any questions.

Excited about their chance for home ownership, the black population lived through the inconveniences “with a nod of mutual understanding.” People selling land knew these were properties that flooded from time to time, but the issue was a mute one because the black community which developed provided a golden opportunity for African-American, urban homeownership in the then highly segregated south of the United States (Kinston Daily Free Press 1976). Rather, what developed was a cultural indifference, a sort of “casual” or intentional amnesia (Klein 2005). From the perspective of those living in the floodplain during the period up to a major event of 1954, this series of minor flood events provided a pool of experiences that led to the implementation of mitigation practices by the local community. This “temporal situatedness” of the risk experience established cultural connections among the residents according to their location in time relative to the hazard events that had occurred up to that point.

A major flood eventually came in 1954 when the extremely fast and damaging Hurricane Hazel hit the city. Hazel was not the first hurricane to strike the North Carolina since 1879, yet the widespread damage it caused stands out (Table 1; Barnes 2001).

**Table 1:** Notorious hurricanes since 1879 within 65 miles of Lenoir County, with overall N.C. deaths and damages. Adapted from Barnes 2001

Name/Date	Category	Maximum wind	Pressure in N.C. (inches)	N.C. deaths	N.C. overall damages (unadjusted)
August 1879	4	168*	No data	40+	No data
September 1883	3	100 + *	No data	53	No data
August 1899	4	140*	No data	25	No data
September 1944	3	110*	27.97	1	$1.5 million
Hazel, 1954	4	150*	27.70	19	$136 million
Ione, 1955	3	107	28.00	7	$88 million
Donna, 1960	3	120*	28.45	8	$25 million
Diana, 1984	2	115	28.86	3	$85 million
Fran, 1996	3	115*	28.17	24	$5.2 billion
Floyd, 1999	2	110*	28.34	52	$6 billion

*Estimated

Hazel came with no evacuation orders, no sirens, and the few Lincoln City locals who had a radio erroneously learned from broadcasts that Hazel was still a long way from the coast (WRAL-TV 2004). Elderly informants described Hazel as the first major impact in living memory, and they perceived the flooding as unusual, with standing water knee-deep for four days. While previous hurricanes had hit North Carolina, they noted that Hazel was the first major hurricane they remembered. They explained that the people of Lincoln City did not know they were living in a “floodplain” until Hurricane Hazel. They mentioned that “even the City of Kinston” did not know the area was a floodplain. “People were buying land and houses,” was the pre-Hazel reality.

Hazel had a significant impact on neighborhood risk perception, as it shifted the temporal frame of reference. The event was referred to by one informant as a “new flood dimension.” Hazel culturally constructed a new temporal reference model that now included a more distinct hazard benchmark with baseline referentiality: an event with relative temporal isolation (“rare”) that became starting point or condition against which future changes were measured (De Vries 2005). Hazel had suddenly and radically shifted the meaning of the landscape: Lincoln City had become a floodplain. After this event, risk became translated into new architectural designs as residents started building houses higher off the ground.

### The Beginning of the Buyout Mitigation Agenda

But while Hazel created shifted baseline flood awareness among residents in Lincoln City, public officials at the City of Kinston level, which had a different flood baseline, nevertheless decided to place a new wastewater treatment plant (“Peachtree”) in the floodplains next to the neighborhood in the early 1960s. When in 1964 extreme rainfall associated with Hurricane Hilda inundated Lincoln City, local officials noted *to their surprise* that their investment needed extra protection. Indeed, officials I spoke with mentioned the 1964 flood, yet rarely spoke of Hazel, and the same trend was found in newspapers (Kinston Free Press 1996d). With shifts in temporal reference models, Hazel and Hilda reduced the vulnerability for city and its residents to these hazards. This is evidenced by the political momentum which the mitigation agenda received soon after 1964, when city officials traveled to the state capital (Raleigh) to lobby for a system of dams that would manage future Neuse River flooding. Further, the momentum created by Hilda helped initiate the first buyout project, which the City of Kinston financed in a neighborhood of Happersville, an area considered “blighted,” and organized by the city’s new Riverway Committee. The committee had developed a restoration plan that included building a greenway and parks along the Neuse River, including Happersville, the downtown area, the Kinston farmers market, and Lincoln City. A former, senior planning official told me how the momentum created by the baseline hazards events led to a window of opportunity for community development:

So really, those two ideas kind of came together. It was an opportunity, like right now you would have found the idea of the much later Acquisition and Relocation Program [FEMA], there were already some fundamental planning blocks, if you will, of ideas as far as creating the park system across the river as a place where it floods, a place probably not the best place to live.

With the same agenda in mind, the city started a $2.1 million Housing and Urban Development financed floodplain acquisition project in Lincoln City in 1978. However, in the 1980s the State of North Carolina changed the criteria for ranking their competitive projects, making elevation and relocation more difficult and the city management reluctantly resorted to more renovation in place.

**Fig. 2:**
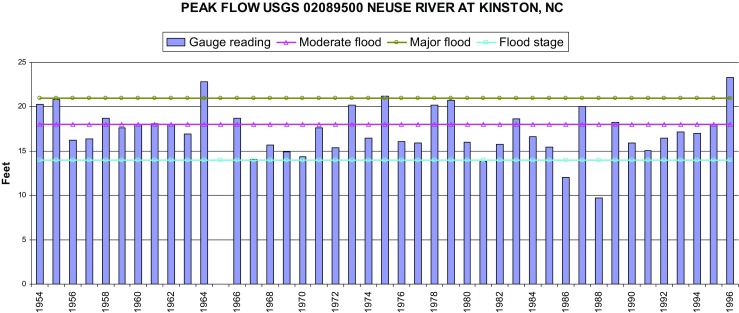
USGS #02089500 Neuse River at Kinston 1964 through 1996

While the mitigation agenda had Lincoln City as one of the target buyout areas, Neuse River heights between 1964 and 1996 never reached major flood levels (except possibly in 1975), although there were certainly a number moderate floods (in 1966, 1972, 1978, 1979, 1983, 1987; Fig. 2).

This period coincided with the eventual building and completion of Falls Lake Dam and Falls Lake Reservoir in 1981. This altered the Neuse River’s flood ecology, apparently reducing flood height levels by managing drainage release from Falls Lake upstream near Raleigh. Although Falls Lake Dam’s operational mandate was not restricted to flood control and included important drinking water and recreational mandates, downstream communities understood its significance to be solely mandated by flood control. After 1981, river heights at Kinston seem to indeed have decreased (Fig. 2). Both residents and the Planning Department readily attributed the reduction in flooding to the dam*.* During this period, the temporal situatedness of the population relative to flood hazards became contextualized not by casual amnesia at the local community scale, but by a city-wide belief in technological progress and temporal reference to two baseline events: 1954 Hurricane Hazel flooding for the residents of Lincoln City, and 1964 rainfall from Hilda for city administrators.

### The Fluke of Fran

A generation later, Hurricane Fran hit Lincoln City on September 6th 1996. When Fran made landfall in Wilmington, Kinston was right on the edge of a swath of four to six inches of rain. Because of the relative lack of recent experience with these events, initial expectations about the impact of the hazard diverged from the reality that followed. In an early report on the impact of the storm, the Kinston Free Press quoted a local Kinston resident who evaluated the impact by referencing Hazel as comparison: “…as bad as Fran was, she was no match for Hazel. That storm took the roof off my house” (Kinston Free Press 1996a). But soon after the immediate wind impact subsided, an early warning was communicated via the Kinston Free Press that high water as a result of heavy rains from Hurricane Fran north of Lenoir County was expected to travel down the Neuse River from Raleigh and could cause flooding in some low-lying areas of Kinston. While the Fire Chief precisely spelled out the area of Lincoln City likely to be affected, the paper provided an optimism shared with the city engineer that there was serious doubt if any of the structures would be affected unless there was additional rain: “We know it is coming … there are only a couple of areas that will be affected. We’re not expecting the Jonestown flood” (Kinston Free Press 1996b). However, on September 11th the newspaper reported not only an additional 1–2 in. of upstream rain, but that in addition State Officials had to deal with rising waters released from dams across the state. Reports from anticipated flooding of homes in upstream Smithfield and Goldsboro were alarming. The newspaper quoted the Goldsboro city manager: “…the expected crest is the highest I’ve ever seen *and I’ve been here 25 years*” (italics added). As the river continued to rise over the following days, the newspaper prepared its subscribers by reviewing past floods. At the same time, the optimistic distancing suggested by the historical review article entitled “Floods a more common occurrence in the past” brought the experiences of the series of pre-1964 floods back to consciousness vividly in context of the developing event. In particular the 1964 Hilda flood became the significant baseline against which to the impact of the coming event was evaluated. In a small article on the evacuation of tobacco warehouses, a floor manager referred the memories of his elder colleagues:

‘They tell me the floor was covered in water when the river flooded in 1964. If it made it in here then, it probably will this year, too.’ The flood of 1964 put the river level up to 22.9 feet. This flood is expected to crest between 23 and 24 feet. ‘This is a new experience to us,’ he said. ‘*We’ve never experienced anything like this, we’re having to pretty much go by what folks at Emergency Management tell us.*’ (Kinston Free Press 1996c) (italics added)

On Monday, September 16th, the Kinston Free Press reported “Neuse river swamps south Kinston” due to a combination of Hurricane Fran, subsequent heavy rains, and releases from the Falls Lake Dam, producing an “unprecedented flood” ( 1996e). Two days later, the Free Press led with an article stating that water would remain high for at least a week, since there were still releases from Falls Lake Dam ( 1996f). Fran came as a surprise, indicating the reintroduction of a vulnerability with temporal origin. Even the floodplain administrator told me he had not thought that Fran could occur:

Well, I have been the floodplain administrator since ‘92. To be honest, from ‘92 to ‘96 I did not think it would ever happen here. It was an area… I was new at the business. Until you see it, you just don’t believe it will happen to you. It was a surprise!

Within a few weeks following the floods, the city organized a public meeting at the Nature Center, located in the area that used to be Happersville, concerning the possibility of a Fran buyout. As Kinston’s former senior planning official explained to me, the idea was to use the history of Happersville as an example of what would be possible in a buyout. This time, the city had taken note of FEMA’s new Hazard Mitigation Grant Program (HMGP) and their attitude was to not waste any time getting started on the buyout, suggesting that the event was seen as a post-disaster “window of opportunity” for a permanent solution of relocating residents out of the floodplain (Kinston Free Press 1996b). As the Free Press reported:

Local leaders say it would be a big gamble—in both dollars and lives—to rebuild hundreds of homes that lie in the Neuse River’s 100 year floodzone. They want residents to consider a buyout from the federal government and moving. ( 1996h)

As a result of the campaign, 420 houses were demolished and residents relocated, preventing an estimated $6.4 million of damage when the next disaster (Floyd) hit. But despite this and Fran’s damaging impact, many residents did not participate, and opposition against a buyout grew. In Kinston, one city official noted that “with Fran the intake people, the consultants, had to go out and walk the streets and knock on doors and send out letters and that sort of thing in order to get people to sign up because there was not any threat of flooding at that time.” I spoke to a senior African-American couple who had participated in the Fran buyout in their new house located on higher ground, who said that there was a lot of bitterness in the community because they felt that there had been no political alternative to moving:

Many people lived there for years and years and years. After Fran [1996], of course, a lot of those people, they did not want to sell. They wanted to really just have their homes redone and they wanted to stay there. …We could stay! They could stay! But what was made clear, if you choose to stay, you were on your own.

These mixed emotions were further aggravated by the historical context in which Fran occurred. Within historical consciousness, Fran challenged the baseline memory-events provided by the Hazel-Hilda scenario that had predicted that a three to four day flood was in the realm of possibility and defined the normal. Fran reoriented this cultural model by suggesting that seven days of slow drainage of flood water was possible, although the peak flow (cubic feet per second) of Fran (24,000) was less than that of Hilda (26,000). But this change in the temporal reference model did not serve to warn people that perhaps underlying, changed ecological conditions should be of greater future concern. Instead, Fran was publicly perceived as a strange event. Although a surprise, to many locals the single event simply did not provide enough evidence of systemic change. Hurricane Fran was interpreted as an accident, or *fluke*. When the buyout manager drove me around in the floodplains of Kinston, he told me that the people did not sign up in 1996 because:

The first one, Fran, they thought was a fluke. One guy who taught my children in school had lived there for 40 years. He was called out by the National Guard. I said why don't you sign up? ‘Well, I've lived here all my life,’ he said, ‘this is not going to happen again.’

Many residents in Lincoln City and Kinston felt it was not Fran that flooded their properties, but human error. Since 1981, Falls Lake Dam had introduced safety to the area, as evidenced by the lack of flooding since Hilda in 1964. As a result, the struggles of the slow draining flood which the city encountered in 1996 were only partly attributed to the hazard itself. Without the interference of the upstream engineers failing to empty the dam before hurricane season—the argument went—the duration of the flood would have been shorter, and to many the flood would have been less dramatic, less or equal to Hilda or Hazel. This sentiment was so prevalent that a local social movement developed in Kinston that included petitions for justice and accusations directed at the U.S. Corps of Engineers (Kinston Free Press 1996g).

The historical context that made Fran appear a fluke may have been echoed in another unsuccessful attempt by the mitigation office to make use of a “window of opportunity” that emerged after rains of El Nino that flooded 200 homes in Rivermond in 1998. The effort remained largely unsuccessful, as it had been during Fran, due, one official argued, to the fact that the city had allowed residents to move back in their homes too soon after the flooding, and that telling them that they “could not build back” appeared more dictatorial than a provision of useful information. With the aid of FEMA, city officials refined their mitigation strategy once again, now finessing the use of the “substantially damaged” designation to lock homeowners in a buyout through federal regulations that made it more difficult to repair or rebuild. The experience further strengthened the resolve of the Planning Department to relocate residents out of the floodplains. The building inspector noted: “We were all committed.”

### The Surprise of Floyd

The arrival of Floyd as a potential threat to the City of Kinston was noted only three days after Hurricane Dennis had made two remarkable journeys over Kinston and left the Neuse River swollen above flood stage on September 11th 1999, cresting at 15 ft (Kinston Free Press 1999a, 1999b). The timing of the monster hurricane Floyd could not have been worse. Suddenly, flooding became a major concern for Lenoir County: “I don’t want to speculate and create a panic,” Lenoir County’s Emergency Manager said, “it’s something we are going to monitor closely and I would encourage residents in the low-lying areas to do the same” (Kinston Free Press 1999c). The paper printed a map of southern Kinston showing the flood boundaries of Hurricane Fran three years earlier. While the amount of rainfall due to Floyd was not known the expectation was that “these same areas could be affected.” One day later, this expectation was confirmed (Kinston Free Press 1999d).

Floyd hit Kinston on Thursday evening with winds of only 75 miles per hour, barely making hurricane strength. While the Friday newspaper reported on the immediate status of Kinston residents, officials communicated that as a result of the large upstream drainage of water through the Neuse River watershed combined with the release of water from Raleigh’s Falls Lake Dam, a delay in flood crest was expected (Kinston Free Press 1999e, 1999f, 1999g, 1999h). In most articles not dealing with the immediate logistics of wind impacts, the overriding sentiment was temporal both in terms of certainty—as in “I have never seen anything like this in my 51 years”—and uncertainty—“I don’t know what it’s going to look like in the morning, God only knows.” At the edge of Lincoln City, where Lincoln and Tiffany streets crossed, the rising flood waters increasingly alarmed residents. Already on Monday September 20, many prepared to leave, but the actual flood height was still not anticipated by many in the neighborhood. The flood turned the small City of Kinston at one point into a virtual island in a sea of washed out roads and swollen creeks, canals, ditches, and tributaries. Many residents were caught in the rising waters after they returned to their homes as they had not been sufficiently warned of the coming floodwaters. The swift river rise surprised enough residents to leave them stranded on the roofs of their houses in the early morning. In total, the city’s emergency services ended up evacuating residents from about 450 flooded homes (Kinston Free Press 1999i).

The city’s buyout manager explained to me how after Floyd, everything changed:

Floyd occurred on a Thursday, I came in this building on Friday. There was nobody here on this end of the building but me. I hear a knock on that window. It was the guy who said he would not sell after Fran. He said, you told me, I did not listen, I am here to tell you I am ready to go. So... in a sense… and this is a … personal observation.. the best thing that ever happened to the City of Kinston with regard to future flood was Floyd. … Because it came almost 3 years to the day, they were both. .. one was September 6, one was September 15, and it made the point that *this can happen to us ANY time*. And therefore we had people, even though both of these projects were volunteers…. they came and signed up, *asked* to be bought out. The mindset changed from a once in forever circumstance to this can happen to us again, and there before we have had that much difficulty. (Italics added)

The mindset had yet again shifted. From a cultural model in which Fran was seen as abnormal—“a fluke”—Floyd undermined the Hazel-Hilda temporal paradigm and showed that Fran in fact had been an early warning. The local buyout manager summarized this new cultural risk model as *temporally indefinite*: “this can happen to us *ANY* time”. Within the mitigation agenda, Floyd’s impact was to combine spatial displacement with temporal “unplacement,” or an inability to make sense out of the temporal situation. The surprise of Floyd created a cognitive crisis of perception. As the senior planning official put it “Floyd made believers out of people.” He suggested it was in particular the influence of Fran, only three years earlier, which made the decisive difference:

And I think that has to do with the repetitive issue. Before Floyd, Hurricane Fran came along. At that time it was a first-time event. Before that it was ‘64, so like 32 years for the previous one, and the persistent idea—what I noticed for the few decades that I was there before the 1996 floods—was “well it happens. But it will be so rare, so… as long as we build outside the 100 year floodplain we should not worry about it [flooding] too much.”

It is within this period of temporal, spatial, and cognitive disorientation that the city’s buyout strategy finally had its decisive, intended impact, achieving nearly 100% success in Lincoln City. In one of the interviews from the buyout study (Fraser *et al.* 2003), the way in which city officials sold the program illustrates how the various elements came together to put flooded property owners in a situation where it would be difficult for them to remain unconvinced of the logic of participation:

We were able to meet them at the center on the edge of the floodplain when they wanted to get to their house. We took them back in boats, whatever it took, to get medicine and clothes…whatever they needed out of their flooded house to where they could use it in a temporary location. At the same time we demonstrated to them how bad it was. We tried to impress the vision of what they were seeing in riding in a boat back to their house and get them to project that out a few years. Are you going to be able or are you going to want to deal with this again? We were able to talk to them. We were able to sell HMGP. We were able to sell our buyout program.

Having learned from previous experiences, the city used legal pressure through the “substantially damaged” designation to create more complications for residents to reject the buyout idea (De Vries and Fraser 2012). In the midst of residents’ chaotic spatial, temporal, and cognitive uncertainty, Floyd provided a rare opportunity for the city to effectively align its planning goals. The city, in fact, added another temporal element to the mix of vulnerabilities, namely the predetermined speed with which it handled the buyout in order to take full advantage of the duration of the crisis—the “window of opportunity.”

## Discussion

As experience dictates, the heightened concern that overrides daily life in a crisis is bound to fade when things return back to “normal.” For any emergency manager this normalcy is the first aim in the alleviation of human suffering. However, as shown in this historical review of 100 years of flood history in a minority neighborhood, the period leading up to the return to normalcy may also be actively manipulated by mitigation managers as a “window of opportunity” for change. In this case history, several critical moments emerged that provided the City of Kinston the opportunity to progressively develop and implement a mitigation buyout program, refining the strategic skills in the process. However, this case shows that social-political explanation of the success of the temporal “opportunity” whereby residents opted to participate despite hesitation, does not fully account for the unprecedented success of the buyout program after Hurricane Floyd. In the light of long-standing resistance and failed attempts, another major factor influencing mitigation success during “windows of opportunity” appeared related to historical vulnerability of the community to surprise, influenced by culturally ascribed referential meaning and learning derived from specific histories of events that can be characterized as a *temporal situatedness*. For example, had Hurricane Hazel occurred earlier in Lincoln City’s colonization of woodlands, the floodplain would have emerged earlier, and black residents might have been able to organize a less casual approach to flooding at an earlier time, perhaps even organizing the construction of protective levees. Had the period between Hurricane Hazel in 1954 and Hurricane Fran in 1996 not been one of low hurricane activity, Fran may have been sufficient to convince more homeowners to accept a buyout offer. Had Fran been followed by Floyd not three but 30 years later, Floyd may have been regarded as another fluke.

**Fig. 3:**
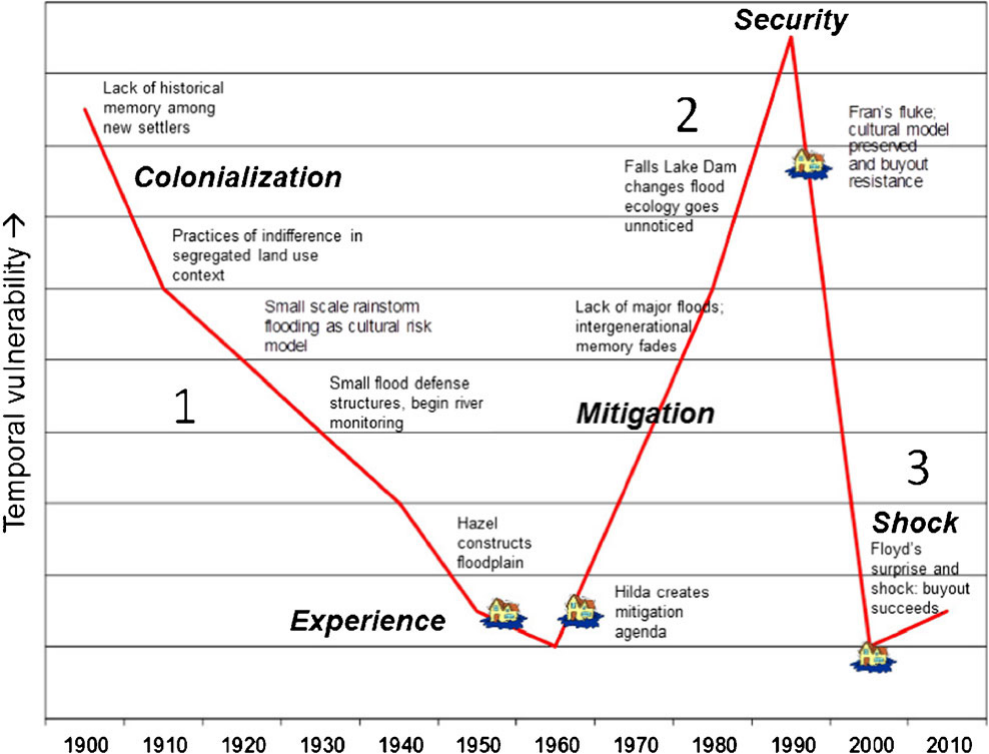
Temporal vulnerability through chronological time with three periods and major influences for residents dwelling in Lincoln City neighborhood

The results show that temporal vulnerability varied historically in at least three distinct periods, each characterized by different temporal situatedness of the population and different critical moments relative to the hazard history (Fig. 3).

This history also emphasizes how this temporal vulnerability is historically produced by the coupling of path-dependent histories with cultural expectations calibrated in cultural memory and other temporal referencing of previous historical events. Four general factors seem to have influenced temporal vulnerability throughout the entire 100 year history described: 1) Epistemological uncertainty of floodplain dynamics, such as is the case in a newly colonized environment or an environment altered as a result of engineering (e.g., a dam); 2) Cultural practices that maintained a casual amnesia, such as a culture of indifference or denial in Linclon City, or optimistic belief in technological progress leading to reduced attention to the primacy of environmental monitoring practices in the city overall; 3) Cultural meaning attributed to stochastic timing of floods, which occur at random moments in time, such as the notion that Hurricane Fran was a fluke, despite the fact that it was a clear warning of systemic change; and 4) Competitive impact of referential baseline attractor—such as Hurricanes Hazel and Hilda—which calibrate the meaning of risk experiences and the salience of historical memory relative to other events. These factors are individually not new to risk analysis. For example, the baseline issue has been long observed in the form of cognitive anchoring bias provided in risk psychology (e.g., Tversky and Kahneman 1974). However, taken together they provide an original analytical framework from which to analyze the extent to which surprise conditions may develop into critical times, and detail a vulnerability perspective that is inherently temporal and may be of influence along with social and physical vulnerabilities commonly studied.

## Conclusion

Using a referential theory I address the concept of a post-crisis “window of opportunity” as a temporal vulnerability that exists because of compromised temporal referentiality embedded in local cultural models. The case study shows how a specific temporal situatedness developed for Lincoln City residents. On the eve of the impact of Hurricane Floyd, this led to a heightened potential for loss—a temporal vulnerability that can be characterized as a condition for collective surprise. This condition materialized as a temporal “unplacement,” or inability to make sense out of the temporal situation. This explanation, I argue, focuses directly on the collective unfreezing of preexisting expectations, and helps to explain why the “window of opportunity” was so successful after Floyd, as opposed, for example, to the “fluke” of Fran. As Floyd’s post-flood “window of opportunity to woo” emerged, mitigation managers successfully obtained high rates of participation. For some residents this was a unique chance to move out. For others it was an unsurmountable barrier to staying in place.
